# Insulin and insulin-like growth factor signaling increases proliferation and hyperplasia of the ovarian surface epithelium and decreases follicular integrity through upregulation of the PI3-kinase pathway

**DOI:** 10.1186/1757-2215-6-12

**Published:** 2013-02-07

**Authors:** Shelby M King, Dimple A Modi, Sharon L Eddie, Joanna E Burdette

**Affiliations:** 1 Department of Medicinal Chemistry and Pharmacognosy, University of Illinois, 900 S. Ashland Room 3202, Chicago, IL, 60607, USA

**Keywords:** Insulin, Insulin-like growth factor, Ovarian surface epithelium, Ovarian follicle

## Abstract

**Background:**

The ovarian surface epithelium responds to cytokines and hormonal cues to initiate proliferation and migration following ovulation. Although insulin and IGF are potent proliferative factors for the ovarian surface epithelium and IGF is required for follicle development, increased insulin and IGF activity are correlated with at least two gynecologic conditions: polycystic ovary syndrome and epithelial ovarian cancer. Although insulin and IGF are often components of *in vitro* culture media, little is known about the effects that these growth factors may have on the ovarian surface epithelium morphology or how signaling in the ovarian surface may affect follicular health and development.

**Methods:**

Ovaries from CD1 mice were cultured in alginate hydrogels in the presence or absence of 5 μg/ml insulin or IGF-I, as well as small molecule inhibitors of IR/IGF1R, PI 3-kinase signaling, or MAPK signaling. Tissues were analyzed by immunohistochemistry for expression of cytokeratin 8 to mark the ovarian surface epithelium, Müllerian inhibiting substance to mark secondary follicles, and BrdU incorporation to assess proliferation. Changes in gene expression in the ovarian surface epithelium in response to insulin or IGF-I were analyzed by transcription array. Extracellular matrix organization was evaluated by expression and localization of collagen IV.

**Results:**

Culture of ovarian organoids with insulin or IGF-I resulted in formation of hyperplastic OSE approximately 4–6 cell layers thick with a high rate of proliferation, as well as decreased MIS expression in secondary follicles. Inhibition of the MAPK pathway restored MIS expression reduced by insulin but only partially restored normal OSE growth and morphology. Inhibition of the PI 3-kinase pathway restored MIS expression reduced by IGF-I and restored OSE growth to a single cell layer. Insulin and IGF-I altered organization of collagen IV, which was restored by inhibition of PI 3-kinase signaling.

**Conclusions:**

While insulin and IGF are often required for propagation of primary cells, these cytokines may act as potent mitogens to disrupt cell growth, resulting in formation of hyperplastic OSE and decreased follicular integrity as measured by MIS expression and collagen deposition. This may be due partly to altered collagen IV deposition and organization in the ovary in response to insulin and IGF signaling mediated by PI 3-kinase.

## Background

The ovarian surface epithelium (OSE) is a single layer of squamous-to-cuboidal cells surrounding the ovary that exhibits both epithelial and mesenchymal characteristics [1]. During monthly ovulations, the primary function of the OSE is to remodel the ovarian surface and underlying extracellular matrix to allow for rupture of a mature follicle. Following oocyte extrusion, the OSE proliferates to heal the wound in the surface of the ovary [2]. OSE have receptors for steroid hormones and growth factors, both of which are found in abundance in follicular fluid released during ovulation [3]. In particular, the OSE has been shown to express insulin receptor (IR) and insulin-like growth factor receptors (IGF1Rs); additionally, at high concentrations insulin can signal through IGF1R or through hybrid receptors of IR and IGF1R [4,5]. Activation of IR or IGF1R by ligand binding activates downstream signaling pathways including the phosphatidylinositiol 3-kinase (PI3K) and mitogen-activated protein kinase (MAPK) pathways. In turn, proliferative and anti-apoptotic pathways are activated, including Akt, glycogen synthase kinase 3 β (GSK3β), Bcl2, and Bad [6].

In immortalized OSE cell lines and many primary cell cultures, insulin is a critical component of the culture medium required for propagation of the cells; however, the ovary is not a classically insulin-responsive tissue [7,8]. Crosstalk can occur between IR and IGF1R signaling when high concentrations of insulin initiate signaling through IGF1R [9]. Interestingly, IGF-I is secreted into follicular fluid by granulosa cells, providing a local source for this cytokine [10]. While it is known that insulin and IGF are proliferative in immortalized OSE cell lines [1], it is unknown whether these growth factors may exhibit additional changes in cell growth when the ovary is cultured in three dimensions (3D). By growing normal OSE as a component of ovarian organoids cultured within alginate hydrogels [11,12], the effects of insulin and IGF on tissue architecture can be determined. The use of alginate hydrogels for organ culture permits growth of tissues in their normal three-dimensional architecture without disruption of signaling pathways downstream of extracellular matrix, as can be observed with other culture materials such as Matrigel [13].

In America, 64% of adult women are considered obese, and this negatively influences reproductive health and fertility [14]. High circulating levels of insulin and IGFs are associated with obesity and diabetes; in the female reproductive system, increased levels of these growth factors are associated with polycystic ovary syndrome (PCOS) and ovarian cancer [15]. For example, PCOS is a leading cause of infertility that affects 5-10% of reproductive-aged women and is diagnosed when patients exhibit at least two of the three following symptoms: anovulation, hyperinsulinemia, and hyperandrogenism [16]. Ovarian cancer is the deadliest gynecological malignancy affecting American women, and obesity and diabetes are associated with a worse prognosis due in part to the effects of elevated levels of insulin and IGF on cancer cells [17,18]. Primary cell lines established from ovarian carcinomas demonstrate that the components of the IGF pathway are present in ovarian cancer, including secreted IGF-I and IGF-II, IGFR-I and IGFR-II, and IGFBPs [19,20]. IGF-II is overexpressed in ovarian cancer cells compared to normal OSE [21]. IGF signaling exerts a pro-proliferative, anti-apoptotic effect on ovarian cancer cells and has also been shown to play a role in mediating cisplatin resistance [22,23].

The current study examines the effects of high insulin and IGF levels on the OSE and ovarian follicles using an alginate hydrogel culture system. High proliferation rates in the OSE following culture with insulin or IGF were observed as described in previous studies [3,24,25]; however, by utilizing a 3D organ culture system, the present study demonstrates that high levels of insulin and IGF induce hyperplasia and formation of multiple cell layers in the OSE. Treatment of organ cultures with the IR/IGF1R inhibitor tyrphostin AG1024 restored the OSE to a single layer of epithelium and reduced proliferation to basal rates. Both the MAPK and PI3K pathways were involved in OSE hyperplasia, as small molecule inhibitors for these pathways inhibited insulin or IGF-induced hyperplasia and proliferation. Upon further examination of ovarian organ cultures, insulin and IGF reduced proliferation of granulosa cells, decreased Müllerian inhibiting substance (MIS) expression, and altered collagen deposition, which were restored upon blockage of IR/IGF1R function with tyrphostin AG1024. In summary, this study highlights the use of a 3D tissue culture system in demonstrating the differential effects that insulin and IGF signaling have on the ovarian surface and follicles.

## Methods

### Animals

CD1 mice were purchased from Harlan (Indianapolis, IN) and experimental animals were acquired through in-house breeding. Animals were treated in accordance with National Institutes of Health Guide for the Care and Use of Laboratory Animals and the established animal care and use protocol at the University of Illinois at Chicago. Animals were housed in a light (12 h light: 12 h dark) and temperature controlled environment and provided food and water *ad libitum*.

### Organ culture

Ovaries from d16 female CD1 mouse pups were used for organ culture experiments. Ovaries were dissected and encapsulated in alginate as described previously [11,12]. The alginate-encapsulated organoids were cultured for 7d in basal medium composed of αMEM (Gibco, Carlsbad, CA), 100 U penicillin (Gibco), and 100 μg/ml streptomycin. DMSO was added at a final concentration of 0.01% (v/v) as a solvent-only negative control. Bovine insulin (Sigma-Aldrich, St. Louis, MO) or recombinant human IGF-I (Ipsen Biopharmaceuticals Inc., Basking Ridge, NJ) was added to cultures at a concentration of 5 μg/ml. AG1024 (Calbiochem, Billerica, MA) was dissolved in DMSO and added at a final concentration of 10 μM. LY294002 (Cell Signaling, Cambridge, MA) was dissolved in DMSO and added at a final concentration of 25 μM. U0126 (Cell Signaling) was dissolved in DMSO and added at a final concentration of 10 μM. Media was changed every four days with fresh growth factors.

### RNA isolation and gene expression analysis

Organoids were cultured for 3d in basal media, 5 μg/ml insulin, or 5 μg/ml IGF-I [11,12]. OSE were collected by treatment with collagenase [12], mRNA was extracted, RNA (0.5 μg) was reverse transcribed using the RT^2^ First Strand kit (Qiagen), and cDNA was added to RT^2^ Profiler PCR Cancer Pathway Finder Arrays (Qiagen) according to manufacturer’s recommendations. Gene expression changes were analyzed on a Viia7 real-time PCR detection system (Applied Biosystems, Carlsbad, CA) and normalized relative to the average expression of β-actin, Gusb, Hprt, Hsp90ab1, and Gapdh according to manufacturer’s instructions.

### Immunohistochemistry

Tissues were prepared for paraffin sectioning and immunohistochemistry or hematoxylin and eosin staining was completed as described previously [26]. Heat-mediated antigen retrieval was performed in 0.1M sodium citrate pH 6.0, followed by blocking with 10% normal serum. Tissue sections were incubated with the following primary antibodies overnight at 4°C: anti-cytokeratin 8 (CK8, 1:100, Developmental Studies Hybridoma Bank, Iowa City, IA); anti-BrdU (1:200, Abcam, Cambridge, MA); anti-Müllerian inhibiting substance (MIS, 1:50, Santa Cruz Biotechnology, Santa Cruz, CA); anti-phospho-glycogen synthase kinase beta (pGSK3β^Ser9^, 1:400, Cell Signaling, Cambridge, MA); total GSK3β (1:100, Cell Signaling); and anti-collagen IV (1:100, EMD Millipore, Billerica, MA). Slides were incubated with biotinylated secondary antibodies, followed by formation of avidin-biotin complexes and detection with 3,3'-diaminobenzidine (Vector Labs, Burlingame, CA). Slides were imaged on a Nikon Eclipse E600 microscope. The percentage of proliferating OSE relative to the total number of OSE was quantified using Image J software (NIH, Bethesda, MD).

### Statistical methods

All data are represented as the standard error of the mean. Statistical analysis was carried out using GraphPad Prism software (GraphPad, La Jolla, CA). Statistical significance was determined by Student’s *t*-test or one-way ANOVA, with *P*< 0.05 considered significant.

## Results

### Insulin and IGF-I induce OSE hyperplasia and multilayering

**Figure 1:**
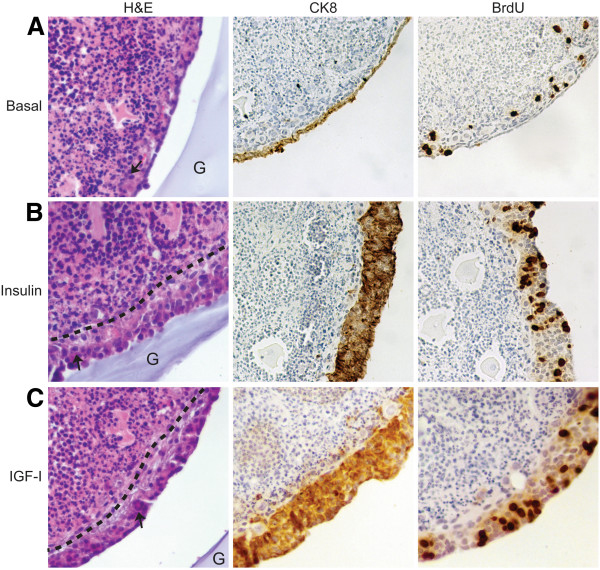
**Insulin and IGF-I induce OSE hyperplasia and proliferation.** Ovarian organoids were cultured in alginate hydrogels in basal media (**A**) or media supplemented with 5 μg/ml insulin (**B**) or IGF-I for 7d (**C**). BrdU was added 24h prior to fixation to label dividing cells. Tissues were stained with hematoxylin and eosin (H&E) to evaluate morphology. Arrow indicates primordial follicle. Dashed line is representative of the field of OSE expansion. Alginate hydrogel stains lightly with hematoxylin and is marked by G. Serial sections were stained with antibodies against cytokeratin 8 (CK8) to mark OSE or antibodies against BrdU to mark dividing cells. All images are 400X.

Culture of ovarian organoids in alginate hydrogels permits analysis of normal OSE growth in the context of its normal microenvironment without the requirement for immortalization with viral antigens [11]. To analyze the effects of specific growth factors on different cell types in the tissue, the culture medium can be supplemented with growth factors, cytokines, steroid hormones, or other factors which are able to diffuse freely across the alginate gel [27]. Organoids were cultured for 7d in basal medium (no serum or growth factors) or medium supplemented with 5 μg/ml insulin or IGF-I. Morphology of the OSE was analyzed by hematoxylin and eosin (H&E) staining or immunohistochemistry for cytokeratin 8 (CK8). To measure proliferation, 5-bromodeoxyuridine (BrdU) was added to the cultures 24h prior to fixation. Organoids cultured in basal medium exhibited a single layer of squamous OSE with few proliferating OSE (Figure 1A). Inclusion of insulin (Figure 1B) or IGF-I (Figure 1C) in the culture medium resulted in formation of a hyperplastic layer of OSE, approximately 4–6 cell layers thick around the outer surface of the ovary. Primordial and primary follicles were frequently observed trapped within this layer of OSE (Figure 1B and C, arrow).

### Insulin and IGF-I induce OSE proliferation in a dose- and time-dependent manner

**Figure 2:**
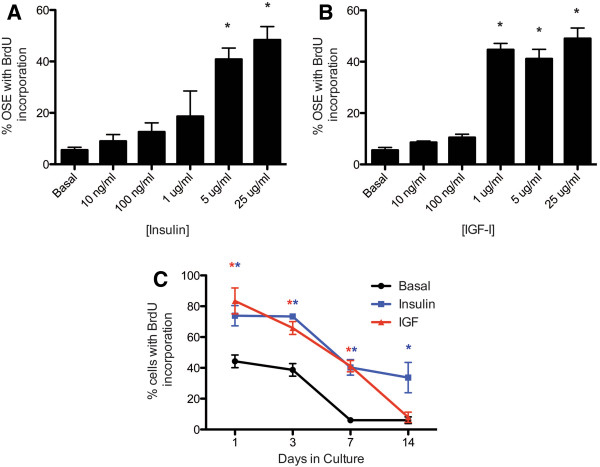
**Insulin and IGF-I increase OSE proliferation in a dose- and time-dependent manner.** Ovarian organoids were cultured for 7d with increasing concentrations of insulin (**A**) or IGF-I (**B**) and analyzed for CK8 and BrdU expression. The percentage of OSE with BrdU incorporation was determined by dividing the number of cells positive for CK8 and BrdU by the total number of CK8-positive cells. **C**, Organoids were cultured for 1, 3, 7, or 14d in basal media (black line), media with 5 μg/ml insulin (blue line), or 5 μg/ml IGF-I (red line) and assessed for the percentage of OSE with BrdU incorporation as described above. All images are 400X. Data shown represent SEM. * indicates *P*<0.05.

To quantify the proliferative effects of insulin and IGF and determine the relative potency of each ligand in the OSE, organoids were cultured for 7d with increasing concentrations of insulin (Figure 2A) or IGF-I (Figure 2A). BrdU was added 24h prior to fixation, and serial sections stained for CK8 and BrdU were analyzed to determine the percentage of proliferating OSE (labeled with BrdU and CK8) relative to the total number of OSE (labeled only with CK8). By d7 of culture, only about 8% of OSE cultured in basal medium were proliferating. Addition of 5 μg/ml insulin or 1 μg/ml IGF-I to the culture medium increased the percentage of proliferating OSE to approximately 41% or 47% respectively, demonstrating that a higher dose of insulin was required to achieve the same proliferative effects of IGF-I. Unless otherwise noted, experiments were completed at 5 μg/ml to reflect the concentration commonly used in media supplements for insulin and a dose that significantly increased proliferation [28,29]. IGF is not commonly used in media and increased proliferation at both 1 and 5 ug/ml, but was used in further experiments at 5 ug/ml to match the concentration of insulin.

The percentage of proliferating OSE was highest at d1 for all treatment groups, with 44% of OSE from organoids cultured in basal media exhibiting proliferation as measured by BrdU incorporation following a 24h label (Figure 2C). Addition of insulin to the media increased this percentage to 74%, and IGF-I increased the percent of proliferating OSE to 83%. The percent of proliferating OSE declined over 14d in culture, but at d3 and d7, OSE cultured with insulin or IGF exhibited increased percentages of proliferating OSE as compared to OSE cultured in basal media. By d14, 34% of OSE cultured with insulin were still proliferating, compared to 8% of OSE cultured with IGF and 6% of OSE cultured in basal medium (Figure 2C).

### Inhibition of IR/IGF1R function restores OSE morphology

**Figure 3:**
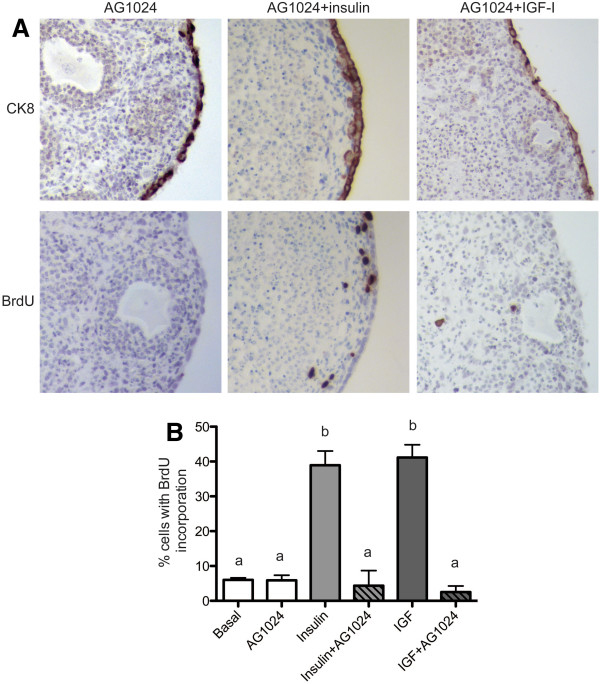
**Inhibition of IR/IGF1R function restores OSE morphology.** Ovarian organoids were cultured 7d with 10 μM AG1024, 10 μM AG1024 plus 5 μg/ml insulin, or 10 μM AG1024 plus 5 μg/ml IGF-I. Tissues were stained with antibodies against CK8 and BrdU (**A**) and the percentage of proliferating OSE was quantified (**B**). All images are 400X. Data shown represent SEM. Statistical differences (*P*<0.05) are between groups labeled a and b.

To validate that signaling through IR or IGF1R mediated OSE hyperplasia and proliferation, the receptor tyrosine kinase inhibitor tyrphostin AG1024, which is a small molecule inhibitor of IR and IGF1R phosphorylation, was incubated with the organ cultures [30]. Culture of ovarian organoids with 10 μM AG1024 alone resulted in a single layer of OSE with 6% of OSE proliferating, which was not statistically different from organoids cultured in basal medium (Figure 3A and B). Addition of AG1024 to media containing 5 μg/ml insulin or IGF-I reduced OSE hyperplasia to a single layer of cells as determined by CK8 staining, which marks the OSE (Figure 3A). AG1024 also reduced insulin-mediated or IGF-mediated proliferation to 4% or 3% respectively (Figure 3B), indicating that the increased proliferation of OSE following culture with insulin or IGF was due to signaling through IR and IGF1R.

### Transcription changes in the OSE in response to insulin or IGF

Few studies have investigated the transcriptional targets downstream of IR/IGF1R signaling in normal OSE. To evaluate changes in gene expression in the OSE following culture with insulin or IGF-I, OSE were collected from organoids after 3d in culture to maximize the possibility of monitoring gene changes occurring as the OSE were undergoing high rates of proliferation and cell growth. Insulin increased expression of insulin-receptor associated proteins, including insulin-like 1 (Insl1; 2.31-fold increase relative to basal) and insulin-like 3 (Insl3; 4.38-fold increase) (Table 1). As evidence of a negative feedback loop, insulin repressed expression of Igfr1 (−2.00) and Igf2 (−2.37). IGF also increased expression of insulin-receptor associated proteins, with a 2.73-fold increase in growth factor receptor-bound protein 10 (Grb10) and a 4.01-fold decrease in Igf2 expression (Table 1). As expected, insulin and IGF both regulated genes involved in metabolism, including an increase in low-density lipoprotein receptor (LDLR: 2.67-fold increase for insulin and 3.81-fold increase for IGF) (Table 1). Gene expression changes downstream of the mitogenic PI3K and MAPK pathways were also evaluated. At the level of transcriptional changes, insulin and IGF repressed subunits of PI3K as well as Akt1 and Akt2 (Table 1). Overall, components of the Ras/Raf pathway downstream of MAPK/Erk were repressed as well by insulin and IGF (Table 1); however, this likely represents negative feedback regulation of the pathway and is not reflective of activated phosphorylated proteins in the signaling cascade.

**Table 1:** Changes in gene expression in the OSE induced by 5 μg/ml insulin or 5 μg/ml IGF-I

**Gene symbol**	**Insulin**	**IGF**	**Gene symbol**	**Insulin**	**IGF**
**Insulin-Receptor associated proteins**			**MAPK Pathway**		
Dok1	−1.24	−4.22	Braf	−1.24	−3.76
Dok2	−2.17	−1.13	Fos	−1.22	2.36
Dok3	−1.79	−14.41	Hras1	−1.04	−2.26
Eif4ebp1	1.63	2.21	Kras	−1.85	3.09
Gab1	−1.17	−2.71	Nos2	−2.11	−21.53
Grb10	1.44	2.73	Rps6ka1	−1.55	−4.44
Igf1r	−2.00	−2.08	Rras	2.07	2.65
Igf2	−2.37	−4.01	Rras2	−1.58	2.55
Igfbp1	1.01	−2.13	Sos1	−1.09	2.80
Insl1	2.31	1.18			
Insl3	4.38	−2.89	**Metabolism**		
Irs2	−1.73	−7.06	Cebpa	1.25	−5.08
Nck1	−1.41	−2.15	Cebpb	1.21	2.48
Ppp1ca	−1.18	−2.83	Fbp1	−2.08	1.10
Ptpn1	−1.05	−3.25	Gpd1	−1.03	−6.32
**PI3K Pathway**			Gsk3b	1.19	2.59
Akt1	−1.26	−4.65	Ldlr	2.67	3.81
Akt2	−1.16	−3.19	Lep	2.14	1.09
Dusp14	−1.93	−3.11	Pparg	1.26	2.25
G6pc	2.31	1.18	Slc27a4	−1.14	−4.55
Hk2	1.75	2.38			
Mtor	−1.38	−3.62	**Other Pathways**		
Pik3ca	−1.21	−2.66	Cfd	1.02	3.10
Pik3r1	−1.49	−3.16	Retn	1.14	−20.16
Pik3r2	−1.16	−3.23	Npy	−1.52	−2.98
Serpine1	3.02	2.66			
Ucp1	2.31	1.18			

Organoids were cultured for 3d in basal media or supplemented with insulin or IGF. RNA was isolated from collected OSE and reverse transcribed for analysis by cDNA transcription array. Data shown represent fold changes relative to insulin (n=2) after normalization to housekeeping genes.

### IGF-I increases pGSK3β signaling in the OSE

**Figure 4:**
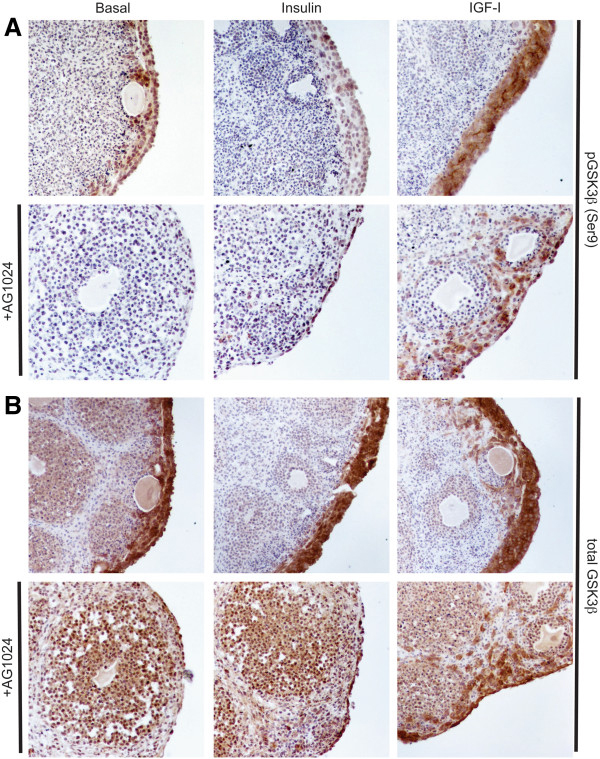
**IGF-I increases pGSK3β signaling in the OSE.** Organoids were cultured 7d in basal media or media containing 5 μg/ml insulin or IGF-I, with or without the addition of 10 μM AG1024. Tissues were stained for expression of GSK3β phosphorylated at Ser9 (**A**) or total GSK3β protein (**B**). All images are 400X.

To validate that changes in PI3K or MAPK signaling occurred along with proliferative changes in the OSE, organ cultures treated with insulin or IGF-I were assessed for phospho-glycogen synthase kinase 3 beta (pGSK3β) and total GSK3β expression by immunohistochemistry. Akt activation induces phosphorylation of GSK3β at serine 9, leading to inhibition of the kinase function of the protein, progression through the cell cycle, and inhibition of apoptotic pathways [31]. From gene expression data, IGF-I induced a 2.59-fold increase in Gsk3b, while insulin induced a 1.19-fold change in Gsk3b (Table 1). Expression of pGSK3β (Ser9) was increased in the OSE of organ cultures treated with IGF-I relative to basal cultures, in agreement with the gene expression data (Figure 4A). This increase in pGSK3βwas redistributed with the AG1024 IR/IGF1R inhibitor into a punctate diffuse pattern; additionally, AG1024 reduced expression of total GSK3β (Figure 4A and B).

### Inhibition of MAPK/Erk signaling reduces insulin-induced OSE hyperplasia

**Figure 5:**
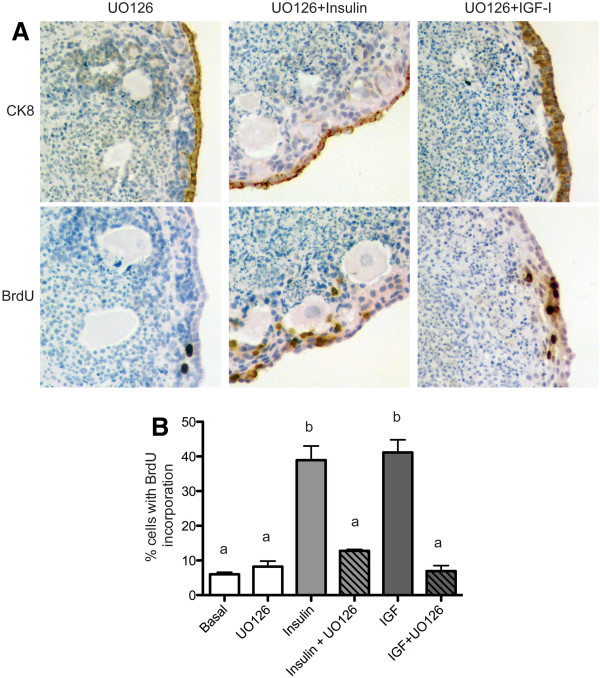
**Inhibition of MAPK/Erk signaling reduces insulin-induced OSE hyperplasia.** Organoids were cultured 7d in media containing the MEK1/2 inhibitor 10 μM UO126, 10 μM UO126 plus 5 μg/ml insulin, or 10 μM UO126 plus 5 μg/ml IGF-I. Tissues were stained for expression of CK8 and BrdU (**A**) and the percentage of proliferating OSE was quantified (**B**). All images are 400X. Data shown represent SEM. Statistical differences (*P*<0.05) are between groups labeled a and b.

Activation of the MAPK pathway is known to occur downstream of IR/IGF1R signaling, leading to increased transcription and cell proliferation [8]. Components of the MAPK pathway were regulated by insulin and IGF in the OSE by transcription array (Table 1). To determine if this signaling pathway was involved in OSE hyperplasia and proliferation, ovarian organoids were cultured with the MEK1/2 inhibitor UO126. When organoids were cultured with UO126 alone, a single layer of OSE was observed with 8% of OSE proliferating, which was similar to organoids cultured in basal media (Figure 5A and B). To determine if inhibition of MAPK signaling by UO126 could reduce the OSE hyperplasia and proliferation induced by insulin, organoids were cultured with both UO126 and insulin. A single layer of OSE was observed, with 13% of OSE proliferating, which was not significantly different from basal rates (Figure 5B). However, organoids cultured with UO126 and IGF-I exhibited several layers of OSE, although the thickness of the OSE was reduced as compared to that induced by IGF-I alone (Figure 5A and Figure 1C). Addition of UO126 to the culture media reduced the percentage of proliferating OSE to 7%, as compared to 41% for IGF-I alone (Figure 5B).

### Insulin- and IGF-induced OSE hyperplasia and proliferation requires PI3K signaling

**Figure 6:**
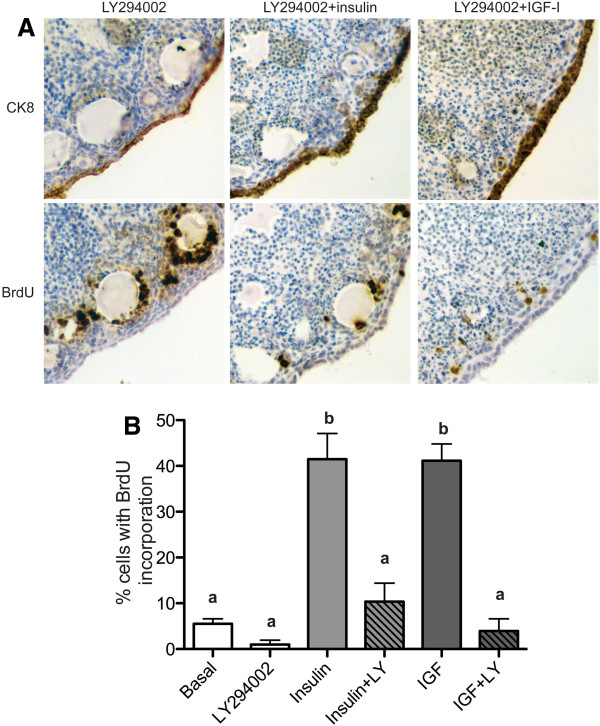
**Insulin- and IGF-induced OSE hyperplasia and proliferation requires PI3K signaling.** Organoids were cultured 7d in media containing the PI3K inhibitor 10 μM LY294002, 10 μM LY294002 plus 5 μg/ml insulin, or 10 μM LY294002 plus 5 μg/ml IGF-I. Tissues were stained for expression of CK8 and BrdU (**A**) and the percentage of proliferating OSE was quantified (**B**). All images are 400X. Data shown represent SEM. Statistical differences (*P*<0.05) are between groups labeled a and b.

Another pathway downstream of IR/IGF1R is the PI3K pathway, which plays a role in cell proliferation, regulation of apoptosis, and directional cell growth [8]. Activation of the PI3K pathway alters orientation of the cytoskeleton through the Rho/Rac/Cdc42 GTPases, as well as affecting other components required for cell polarity and migration [32]. Targets of the PI3K pathway were altered in response to insulin and IGF (Table 1) and the OSE exhibited altered morphology, hyperplasia, and multilayering in response to insulin and IGF, indicating that activation of the PI3K pathway may be involved in this phenotype. Organoids cultured with 10 μM LY294002, a PI3K inhibitor, exhibited a single layer of OSE with only 1% of OSE proliferating (Figure 6A and B). To determine if LY294002 could effectively block insulin- or IGF-induced hyperplasia and proliferation, organoids were cultured with LY294002 and insulin or IGF. Culture of organoids with insulin plus LY294002 or IGF-I plus LY294002 resulted in growth of a single layer of OSE (Figure 6A), unlike organoids cultured with UO126, which only completely blocked insulin-induced OSE hyperplasia (Figure 5A). LY294002 reduced insulin-induced OSE proliferation from 41% to 10%, and reduced IGF-induced OSE proliferation from 41% to 4% (Figure 6A).

### High levels of insulin and IGF-I decrease secondary follicle MIS expression

**Figure 7:**
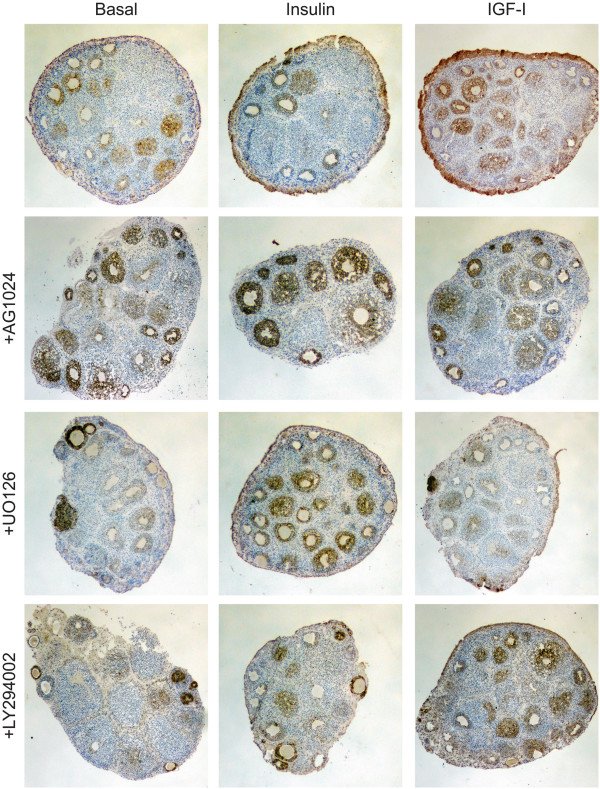
**High levels of insulin and IGF-I decrease secondary follicle MIS expression.** Organoids were cultured for 7d in the indicated media. Tissues were stained for expression of MIS. All images are 100X.

In the mouse ovary, immature primordial and primary follicles are located in the cortex close to the surface of the ovary, with maturing follicles found in the medulla and perimedullary zone [33]. As follicles become activated and begin to mature into secondary and preantral follicles, granulosa cells proliferate to form multiple cell layers around the oocyte and begin to secrete Müllerian Inhibiting Substance (MIS) [33]. IGF secreted by granulosa cells is required for follicle maturation beyond the antral stage [10]; however, high levels of insulin or IGF can be detrimental to follicle development, resulting in polyovular follicles, ovarian cysts, and poor oocyte quality [34-36]. To determine if insulin or IGF affected the follicles as well as the OSE, the expression of MIS by the secondary follicles was analyzed. All organoids exhibited localization of MIS to the ovarian surface as expected, with organoids cultured with insulin or IGF exhibiting several cell layers of OSE expressing MIS, providing a second marker indicating expansion of this cell type in response to insulin and IGF signaling (Figure 7). Secondary follicles were classified morphologically based on the appearance of at least 2 layers of granulosa cells surrounding the oocyte. In basal-cultured organoids, most secondary follicles exhibited MIS expression; however, addition of insulin or IGF to the culture media resulted in reduced expression of MIS in secondary follicles, which could be rescued by addition of tyrphostin AG1024 to the media to block IR and IGF1R signaling (Figure 7). Inhibitors of the MAPK and PI3K pathway did not equivalently restore MIS expression following treatment with insulin or IGF-I, as culture of organoids with UO126 restored MIS expression when organoids were cultured with insulin, but LY294002 restored expression of MIS when organoids were cultured with IGF-I.

### Culture of organoids with insulin or IGF-I disorders collagen-IV organization

**Figure 8:**
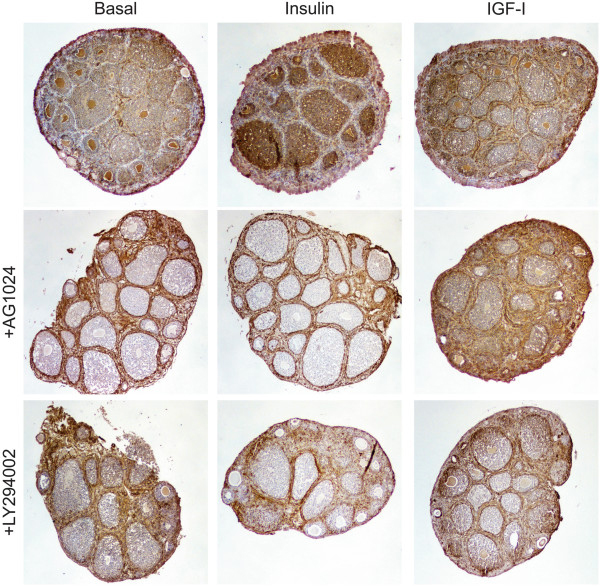
**Culture of organoids with insulin or IGF-I disorders collagen-IV organization.** Organoids were cultured for 7d in the indicated media. Tissues were stained for expression of collagen IV. All images are 100X.

Inclusion of high levels of insulin or IGF-I in ovarian organoid culture medium resulted in hyperplastic OSE and reduced follicle MIS expression (Figure 1 and Figure 7). Recent work suggests that the mechanical forces within the ovary may be involved in follicle maturation and ovulation [37]. Expression of extracellular matrix proteins (ECM) in the ovary has been well characterized, with collagen IV expressed abundantly in the OSE and theca cells, with very low levels in the granulosa cells and stroma [38]. To determine if culture of organoids with insulin or IGF-I resulted in altered ECM deposition or organization, organoids were analyzed for localization of collagen IV. Organoids cultured in basal medium exhibited strong expression of collagen IV in the OSE and theca, but collagen IV was also detected in the granulosa cells (Figure 8). Addition of insulin to the medium resulted in a dramatic increase in collagen IV expression in the granulosa cells, with little expression observed in the theca. Organoids cultured with IGF-I exhibited a similar expression pattern as basal-cultured organoids, with collagen IV expressed primarily in the OSE and theca, with low expression in the granulosa cells. Abrogation of IR and IGF1R signaling by AG1024 alone altered the deposition of collagen such that the follicles were surrounded with collagen and very little expression was detected in the granulosa cells which was a phenotype that resembled uncultured ovaries and was different than basal organs. The resulting phenotype from AG1024 alone suggested antagonizing endogenous IGF resulted in collagen deposition more similar to uncultured ovaries. AG1024 in combination with insulin also resulted in collagen IV expression restricted to the OSE and theca, resembling normal, uncultured ovaries [38]. However, addition of AG1024 to organoids cultured with exogenous IGF did not alter the collagen IV distribution back to resembling uncultured ovaries, suggesting that 10 μM of the inhibitor could not effectively block all the endogenous and exogenous IGF. Although inhibition of MAPK by UO126 did not rescue collagen IV localization (data not shown), inhibition of the PI3K pathway by LY294002 reduced granulosa cell expression of collagen IV to those of organoids cultured with AG1024 alone, indicating that the PI3K pathway may play a central role in altered collagen synthesis and deposition downstream of insulin and IGF signaling (Figure 8).

## Discussion

*In vitro* culture of primary human or mouse OSE often requires inclusion of insulin in the media to induce proliferation [7,39]. Although insulin and the related growth factor IGF-I have been shown to alter epithelial polarity and directional cell growth [32], little is known about how these growth factors may affect directional growth of the OSE. Normal OSE grows on the outer surface of the ovary as a single layer of squamous-to-cuboidal epithelium; however, at concentrations routinely used for culture of primary cells, insulin and IGF-I induced formation of hyperplastic OSE 4–6 cell layers thick likely due to a dramatic increase in the percentage of OSE undergoing proliferation (Figures 1 and 2). Importantly, the concentrations used in the present study and in typical cell culture media are higher than circulating levels or levels found in follicular fluid. Physiological concentrations in the ovary range from 0.5-10 ng/mL insulin and 100-500 ng/mL IGF [40]. Previously IGF1 at 100 ng/mL was reported to increase OSE proliferation [3,41]. The signaling pathway primarily responsible for this hyperplasia was the PI3K pathway, as inclusion of the PI3K inhibitor LY294002 restored growth of the OSE to a single cell layer (Figure 6). The PI3K pathway plays an important role in cell polarity through regulation of the actin cytoskeleton. Activation of PI3K at the plasma membrane in turn leads to activation of Akt, which plays a critical role in chemotaxis and migration of many normal as well as cancerous cell types [42]. Activation of this pathway may also repress expression of E-cadherin, a component of the epithelial cell tight junction that functions to establish and maintain cell polarity that is often altered in ovarian cancer cells to permit increased metastasis [43]. While no universally accepted precursor lesion exists for ovarian cancer originating in the OSE, menopausal ovaries and some mouse models of ovarian cancer exhibit hyperplasia of the OSE, formation of papillary structures, and inclusion cysts [44,45]. Insulin and IGF-I did not induce transformative changes in OSE as measured by growth in soft agar (data not shown); however, it is possible that if levels of insulin and IGF accumulate enough locally in disease they might act on early stages of ovarian cancer to increase proliferation and alter cell polarity to encourage hyperplasia.

The OSE is able to secrete its own ECM, which may play a role in wound healing following ovulation [46]. In particular, OSE express collagen I and collagen IV in the basement membrane that delineates the OSE from the stroma [38]. Since insulin and IGF-I induced formation of hyperplastic OSE (Figure 1), the effects of insulin and IGF-I on collagen IV expression and localization were analyzed to determine if the hyperplasia included changes in cell polarity. Organoids cultured in basal media exhibited strong collagen expression in the OSE and theca cells as expected, with low levels observed in the granulosa cells (Figure 8). However, insulin dramatically increased collagen IV expression in the granulosa cells, which may correlate with reduced expression of MIS in secondary follicles (Figure 7). Inhibition of IR/IGF1R function with tyrphostin AG1024 resulted in collagen IV expression restricted to the OSE and theca as well as increased MIS expression in granulosa cells (Figures 7 and 8). Studies from the Woodruff lab have demonstrated that altered cortical rigidity can disrupt folliculogenesis, as a more rigid environment favors androgen secretion and reduced follicle growth [27,37]. As high levels of insulin cause hyperplastic OSE and increased collagen deposition in the OSE and granulosa cells, this may possibly increase cortical tension on the ovarian follicles to restrict their growth and reduce MIS expression. The detrimental effects of high levels of insulin or IGF on follicle growth may be also be mediated directly by increased MAPK and PI3K signaling.

The MAPK and PI3K pathways are canonical signaling pathways downstream of IR and IGF1R activation [6]. Ovarian organoids cultured with inhibitors of the insulin/IGF pathway appeared to have more MIS expression in the granulosa cells indicating that the ovary has endogenous production of IGF that in *ex vivo* 3D culture is detrimental to the tissue. In the current study, inhibition of the MAPK pathway more effectively blocked insulin-induced OSE hyperplasia and follicular degeneration and was less effective at attenuating the effects of IGF-I. When the MAPK inhibitor UO126 was included along with insulin in the culture medium, the OSE grew as a single layer of cells and the secondary follicles produced MIS (Figures 5 and Figure 7). However, collagen IV expression was still detected in the granulosa cells (data not shown), indicating that additional signaling pathways may be involved in the process of altered ECM deposition in response to insulin. The PI3K inhibitor LY294002 effectively reduced OSE multilayering and proliferation induced by either insulin or IGF-I (Figure 6) as well as restoring MIS expression (Figure 7). This correlated with expression of collagen IV being restricted to the OSE and theca cells similar to when organoids were cultured with the IR/IGF1R inhibitor AG1024 (Figure 8), indicating that PI3K signaling may control collagen IV synthesis or deposition in the ovary, although future work is necessary to delineate the role of each of these pathways in the OSE.

Use of an alginate hydrogel 3D culture system facilitates observation of how different cell types in the ovary interact with one another when stimulated with insulin or IGF-I. As an example, IGF-I is produced locally from the granulosa cells [10] and may be responsible for the low levels of collagen IV observed in basal-cultured organoids (Figure 8) while inhibition of endogenous IGF signaling by AG1024 was able to restore collagen to the appearance of uncultured ovaries. It is unknown whether high levels of insulin and IGF directly or indirectly affect follicle health through expansion of the OSE, resulting in a restricted growth environment as all conditions that enhanced MIS expression also reduced OSE multilayering. Increased collagen deposition has been observed in the stroma of PCOS patients [47] and although PCOS is a complex syndrome involving many different tissues type, this culture system provides an interesting new model of chronic exposure to insulin and IGF that resulted in a thickened ovarian surface layer and aberrant collagen deposition that could impede follicular rupture.

## Conclusions

In this study, an alginate hydrogel culture system was used to investigate the effects of high levels of insulin and IGF-I on normal ovarian surface epithelium. Insulin and IGF-I induced OSE proliferation and hyperplasia resulting in formation of multiple cell layers of OSE, which could be reversed by inhibition of the PI3K pathway. Granulosa cell health as assessed by MIS expression was reduced following culture of organoids with insulin or IGF-I. Inhibition of the MAPK pathway effectively restored MIS expression in organoids cultured with insulin, while inhibition of PI3K signaling restored increased MIS expression in organoids cultured with IGF-I. Therefore, the OSE responds to insulin and IGF-I by proliferating and altering the deposition of collagen, which cannot be discerned in traditional 2D systems. By culturing the ovarian surface in three-dimensions with the stroma and ovarian follicles intact, a new phenotype was discovered suggesting that high levels of insulin and IGF signaling promote hyperplasia of the ovarian surface and encourage changes in collagen deposition that impair granulosa cell function.

## Abbreviations

BrdU: Bromodeoxyuridine; CK8: Cytokeratin 8; ECM: Extracellular matrix; GSK3β: Glycogen synthase kinase 3β; IGF: Insulin-like growth factor; IGFBP: Insulin-like growth factor binding protein; IGF1R: Insulin-like growth factor receptor; IR: Insulin receptor; MAPK: Mitogen activated protein kinase; MIS: Müllerian inhibiting substance; OSE: Ovarian surface epithelium; PCOS: Polycystic ovary syndrome; PI3K: Phosphatidylinositol 3-kinase.

## Competing interests

The authors declare that they have no competing interests.

## Authors’ contributions

SMK carried out the organ culture experiments, immunohistochemical analyses and microscopy, and drafted the manuscript. DAM performed analysis of the effects of UO126 by immunohistochemistry. SLE performed transcription arrays. JEB participated in the design of the study and preparation of the manuscript. All authors have read and approved the final manuscript.
